# Correlation Between Hashimoto's Thyroiditis–Related Thyroid Hormone Levels and 25-Hydroxyvitamin D

**DOI:** 10.3389/fendo.2020.00004

**Published:** 2020-02-14

**Authors:** Guanqun Chao, Yue Zhu, Lizheng Fang

**Affiliations:** Department of General Practice, Sir Run Run Shaw Hospital, Zhejiang University, Hangzhou, China

**Keywords:** hashimoto's thyroiditis, 25-hydroxyvitamin D [25(OH)D], free triiodothyronine (FT3), thyroxine (FT4), thyroid-stimulating hormone (TSH)

## Abstract

**Objective:** The purpose of this study was to further clarify the association of Hashimoto's thyroiditis (HT) and vitamin D deficiency, and to seek the connection between them and related influencing factors.

**Methods:** Data were obtained from subjects who underwent health examinations from January 2018 to December 2018. The diagnosis of HT was based on: antithyroid peroxidase antibody (TPO-Ab) levels >35 IU/ml and/or antithyroglobulin antibody (Tg-Ab) levels >40 IU/ml. Based on the Endocrine Society guidelines, 25-hydroxyvitamin D [25(OH)D] levels ≥30.0 ng/ml were classified as a vitamin D sufficiency; those between 20 and 29.9 ng/ml, as an insufficiency; and those <20 ng/ml, as a deficiency. All statistical analysis was performed by software R.

**Results:** Of a total of 75,436 individuals who were physically examined, 5,656 of them had 25(OH)D levels tested at the same time; 5,230 were enrolled. The level of 25(OH)D in the non-HT group was higher than that in the HT group. Multiple regression analysis showed that HT was statistically significantly correlated with being male, body mass index (BMI), waist circumference, and thyroid-stimulating hormone (TSH). TSH levels in the insufficiency group and deficiency group were higher than those in the sufficiency group. Free triiodothyronine (FT3) and thyroxine (FT4) levels in the insufficiency group and deficiency group were lower than those in the sufficiency group. 25(OH)D increased by 1 ng/ml at the normal reference level, with an increase of 2.78 ng/dl in FT4 concentration and a decrease of 0.17 mIU/L in TSH.

**Conclusions:** Patients with HT present with a reduced 25(OH)D level, and TSH is an independent risk factor for HT. TSH is negatively correlated with 25(OH)D level. FT3 and FT4 levels were positively correlated with 25(OH)D levels.

## Introduction

Hashimoto's thyroiditis (HT) is a chronic autoimmune thyroiditis accompanied by lymphocytic infiltration, which may ultimately lead to destruction of thyroid tissue (1). Some studies suggest that HT is the result of the combined action of genetic susceptibility and environmental factors, but the exact mechanism is still unclear (2). Clinical studies have suggested that HT is associated with the occurrence of papillary thyroid carcinoma (3). One study suggested that in normal thyroid function, HT can also cause neuroinflammation, leading to emotional alterations (4). At present, the incidence of HT is also increasing; most patients do not show symptoms, often found in the physical examination. Because patients may have abnormal thyroid function in the later stage, and may be associated with other thyroid diseases or even malignant tumors, it also brings a psychological and economic burden to patients. Therefore, understanding the incidence of HT in the healthy population and its relationship with other relevant indicators is conducive to in-depth study of HT.

Some studies have suggested that vitamin D has protective effects on autoimmune thyroid diseases and thyroid malignancies, but the mechanism has not been clarified (5). Serum levels of 25-hydroxyvitamin D [25(OH)D] can reflect the nutritional status of the whole body and are used as an indicator of whether vitamin D is adequate in the body (6). A growing body of research supports the important role of adequate vitamin D in health. Vitamin D has also been found to be associated with a variety of inflammation, and supplementation with vitamin D is beneficial in reducing inflammation (7). The researchers found that vitamin D deficiency was seen in all races and in all age groups (8). Vitamin D deficiency is more common in obese people or obesity-related diseases, such as diabetes, so vitamin D supplements may also be a potential treatment (9). Vitamin D levels are not currently a concern for healthy people. The purpose of this study was to further investigate the association between HT and vitamin D deficiency by collecting data from healthy subjects and using retrospective analysis.

## Methods

### Data and Methods

**Figure 1:**
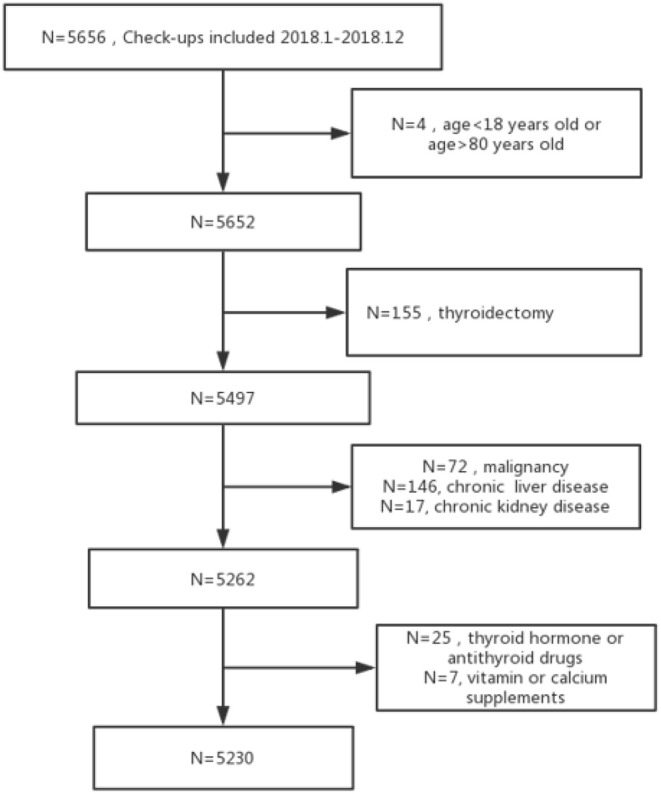
Flowchart of the study population.

Data were obtained from subjects who underwent health examination in the health promotion center of Sir Run Run Shaw Hospital of Zhejiang University from January 2018 to December 2018 (Figure 1). Inclusion criteria: subjects who underwent the medical history questionnaire, abdominal ultrasonography, and laboratory tests of thyroid function and antithyroid antibody, as well as serum 25(OH)D levels. Exclusion criteria: (1) age <18 years or age > 80 years; (2) patients with history of thyroidectomy; (3) patients with other autoimmune diseases; (4) patients with malignant tumors; (5) patients with chronic kidney or liver diseases; (6) users of thyroid hormones or antithyroid drugs or vitamin or calcium supplements

### Clinical and Laboratory Assessments

Height and body weight were measured using a digital scale, and body mass index (BMI) was calculated as body weight (kg)/height squared (m^2^). Waist circumference was measured at the umbilical level by a well-trained nurse. Systolic blood pressure and diastolic blood pressure were measured after 15 min rest. The blood sample for laboratory tests was drawn from individuals after 8 h or more of fasting, and tests included the following: serum fasting blood glucose (FBG), total cholesterol (TC), triglycerides (TG), low-density-lipoprotein cholesterol (LDL-C), high-density-lipoprotein cholesterol (HDL-C), creatinine, and uric acid (UA); serum calcium, albumin, and phosphorus were measured using a Hitachi 7600 clinical analyzer (Hitachi, Tokyo, Japan). Serum thyroid-stimulating hormone (TSH), thyroxine (FT4), free triiodothyronine (FT3), thyroid peroxidase antibody, (TPO-Ab) and thyroid globulin antibody (Tg-Ab) were quantified using chemiluminescent enzyme immunoassays (ICMA; Abbott, Chicago, IL, USA). Serum 25(OH)D concentration was measured with radioimmunoassay and automeasured by using a Roche cobas 8000 automatic biochemical analyzer.

### Diagnostic Criteria

The diagnosis of HT was based on the laboratory test findings: antithyroid peroxidase antibody (TPO-Ab) levels >35 IU/ml and/or antithyroglobulin antibody (Tg-Ab) levels >40 IU/ml. Based on the Endocrine Society guidelines, 25(OH)D levels ≥30.0 ng/ml were classified as a vitamin D sufficiency; those between 20 and 29.9 ng/ml, as an insufficiency; and those <20 ng/ml, as a deficiency.

### Statistical Analysis

All statistical analysis was performed by software R (Version 3.5.1) for Windows. Comparisons of continuous variables between the two groups were performed with the Student's *t*-test or Mann–Whitney *U* test, and categorical variables were compared using the chi-square test. One-way ANOVA and Tukey's *post hoc* test were used to identify the significant differences among the sufficiency, insufficiency, and deficiency groups of 25(OH)D. Parametric correlations were performed by using Pearson's test, and the correlations between 25(OH)D and other variables were calculated using multiple linear regression models. Variables that were statistically significant by univariate analysis and known risk factors were added to a multiple logistic regression model to identify independent predictors of the presence of HT. The mean value of continuous data is expressed as mean ± SD. *P* < 0.05 were considered statistically significant.

## Results

### General Data and Correlation Analysis

There were a total of 75,436 physical examinees from January 2018 to December 2018. Only 5,656 of them had 25(OH)D levels tested at the same time. A total of 5,230 subjects were finally enrolled. The mean age was 48.95 ± 9.06 years old, and 60.1% were male. Clinical and laboratory characteristics are shown in Table 1. BMI, waist circumference, systolic blood pressure, diastolic blood pressure, blood glucose, TG, HDL, alanine aminotransferase (ALT), aspartate aminotransferase (AST), UA, and TSH were significantly different between the non-HT group and the HT group (*p* < 0.05). The level of 25(OH)D in the non-HT group was higher than that in the HT group (*p* = 0.014). Vitamin D deficiency was prevalent in 72.0% of the non-HT group and 76.1% of the HT group, with no statistically significant difference (*p* = 0.238).

**Table 1:** Characteristics of subjects stratified according to non-HT or HT.

	**Non-HT (*n* = 4,889)**	**HT (*n* = 373)**	**Overall (*n* = 5,712)**	**^a^*P*-value**
Male gender, %	3,038 (62.1%)	125 (33.5%)	3,163 (60.1%)	<0.001
Age, years	48.99 ± 9.04	48.51 ± 9.36	48.95 ± 9.06	<0.001
BMI, kg/m^2^	24.37 ± 3.30	23.76 ± 3.14	24.32 ± 3.30	<0.001
WC, cm	85.27 ± 10.58	81.07 ± 10.04	84.97 ± 10.60	<0.001
**BLOOD PRESSURE**				
SBP, mmHg	121.42 ± 15.74	117.88 ± 16.69	121.17 ± 15.84	<0.001
DBP, mmHg	72.34 ± 11.11	69.31 ± 11.30	72.13 ± 11.15	<0.001
FBG, mmol/L	5.45 ± 1.25	5.36 ± 1.17	5.45 ± 1.25	0.141
TG, mg/dl	1.86 ± 1.54	1.69 ± 1.24	1.84 ± 1.52	0.012
TC, mg/dl	4.75 ± 0.93	4.72 ± 0.89	4.75 ± 0.93	0.602
HDL-C, mg/dl	1.17 ± 0.29	1.21 ± 0.29	1.17 ± 0.29	0.011
LDL-C, mg/dl	2.59 ± 0.72	2.58 ± 0.71	2.59 ± 0.72	0.763
ALT, IU/L	26.36 ± 22.90	21.01 ± 12.91	25.98 ± 22.38	<0.001
AST, IU/L	20.39 ± 11.30	18.77 ± 6.87	20.27 ± 11.05	<0.001
UA, μmol/L	363.57 ± 90.40	329.22 ± 80.52	361.14 ± 90.16	<0.001
TSH, mIU/L	1.73 ± 1.03	2.38 ± 2.00	1.78 ± 1.14	<0.001
FT3, pg/ml	2.77 ± 0.33	2.72 ± 0.70	2.77 ± 0.37	0.156
FT4, ng/dl	1.54 ± 0.82	1.51 ± 0.89	1.54 ± 0.82	0.528
25(OH)D, ng/ml	16.66 ± 6.51	15.81 ± 6.42	16.60 ± 6.51	0.014
25(OH)D classification				0.238
Sufficiency	151 (3.1%)	10 (2.7%)	161 (3.1%)	
Insufficiency	1,219 (24.9%)	79 (21.2%)	1,298 (24.7%)	
Deficiency	3,519 (72.0%)	284 (76.1%)	3,803 (72.3%)	

*HT, Hashimoto's thyroiditis; BMI, body mass index; SBP, systolic blood pressure; DBP, diastolic blood pressure; WC, waist circumference; FBG, fasting blood glucose; UA, uric acid; TC, total cholesterol; TG, triglycerides; LDL-C, low-density-lipoprotein cholesterol; HDL-C, high-density-lipoprotein cholesterol; ALT, alanine aminotransferase; AST, aspartate aminotransferase; TSH, thyroid-stimulating hormone; FT3, free triiodothyronine; FT4, thyroxine; 25(OH)D, 25-hydroxyvitamin D*.

a *Two-sided P-values for the difference between the non-HT groups and HT groups, based on chi-squared test for sex and 25(OH)D classification, and independent t-tests for other variables*.

### Independent Factor Analysis of HT

In the univariate regression analysis, HT patients were positively correlated with being male, BMI, systolic blood pressure, diastolic blood pressure, waist circumference, TG, TSH, FT3, and 25(OH)D. After adjusting for known risk factors, multiple regression analysis showed that HT was statistically significantly correlated with being male, BMI, waist circumference, and TSH, but not with 25(OH)D (OR 0.996, 95% CI 0.979–1.013, *p* = 0.689, Table 2), indicating that 25(OH)D was not independently correlated with increased prevalence of HT.

**Table 2:** Odd ratios of HT prevalence by demographic and metabolic factors.

	**Univariate**		**Multivariate**	
**Exposure**	**OR (95% CI)**	***p*-value**	**OR (95% CI)**	***p*-value**
Male gender	0.307 (0.245–0.383)^a^	<0.001	0.439 (0.321–0.602)^a^	<0.001
Age	0.994 (0.983–1.006)	0.333		
BMI	0.943 (0.912–0.975)^a^	<0.001	1.099 (1.025–1.178)^a^	0.008
SBP	0.986 (0.980–0.993)^a^	<0.001	1.002 (0.991–1.013)	0.732
DBP	0.976 (0.967–0.985)^a^	<0.001	0.988 (0.971–1.004)	0.143
WC	0.961 (0.951–0.971)^a^	<0.001	0.961 (0.937–0.986)^a^	0.002
FBG	0.932 (0.839–1.023)	0.166		
TG	0.905 (0.820–0.986)^a^	0.034	1.008 (0.916–1.091)	0.856
TC	0.971 (0.865–1.087)	0.614		
HDL-C	0.978 (0.843–1.131)^a^	0.012	0.702 (0.455–1.075)	0.107
LDL-C	1.339 (1.308–1.370)	0.766		
TSH	1.389 (1.297–1.493)^a^	<0.001	1.335 (1.242–0.436)^a^	<0.001
FT3	0.629 (0.451–0.870)^a^	0.006	1.063 (0.769–1.402)	0.692
FT4	0.956 (0.837–1.087)	0.497		
25(OH)D	0.979 (0.963–0.996)^a^	0.014	0.996 (0.979–1.013)	0.689

*^a^Two-sided P values for the difference between the non-HT groups and HT groups, based on chi-squared test for sex and 25(OH)D classification, and independent t-tests for other variables*.

### The Correlation Factor Analysis of the Vitamin D Group

**Figure 2:**
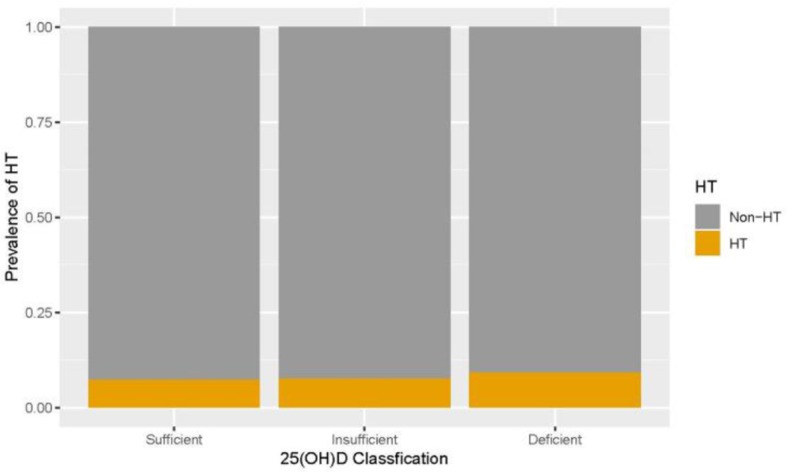
The prevalence of Hashimoto's thyroiditis (HT) after stratification according to 25-hydroxyvitamin D [25(OH)D] classification.

Because multivariate logistic regression showed that 25(OH)D was not associated with the prevalence of HT, we further analyzed the prevalence of stratified HT according to the 25(OH)D classification. The ANOVA comparison of mean values between the three groups showed significant differences in age, BMI, waist circumference, systolic blood pressure, diastolic blood pressure, fasting glucose, TC, TG, LDL, UA, FT3, and FT4 (*p* < 0.05, Table 3). There were significant differences in thyroid hormones among the three groups. TSH levels in the insufficiency group and deficiency group were higher than those in the sufficiency group (*F*_(3.72)_, *p* = 0.024). FT3 levels in the insufficiency group and deficiency group were lower than those in the sufficiency group [*F*_(8.34)_, *p* < 0.001]. In addition, FT4 levels in the insufficiency group and deficiency group were lower than those in the sufficiency group [*F*_(220.4)_, *p* < 0.001] (Table 3). There was no significant difference in the prevalence of HT among the different 25(OH)D level groups (*p* = 0.186) (Figure 2).

**Table 3:** The prevalence of HT and characteristics of subjects stratified according to 25(OH)D classification.

	**Sufficiency (*n* = 161)**	**Insufficiency (*n* = 1,298)**	**Deficiency (*n* = 3,803)**	***P*- value**
Male gender, %	112 (69.6%)	899 (69.3%)^b^	2152 (56.6%)^c^	<0.001
Age, years	52.45 ± 8.83	50.63 ± 8.79^b^	48.23 ± 9.06^c^	<0.001^*^
BMI, kg/m^2^	24.27 ± 3.10	24.72 ± 3.06^b^	24.19 ± 3.37	<0.001^*^
WC, cm	85.34 ± 8.97	86.67 ± 9.61^b^	84.37 ± 10.91	<0.001^*^
**BLOOD PRESSURE**				
SBP, mmHg	124.75 ± 13.16	123.53 ± 15.58^b^	120.22 ± 15.92^c^	<0.001^*^
DBP, mmHg	73.94 ± 10.14	73.52 ± 10.91^b^	71.58 ± 11.22^c^	<0.001^*^
FBG, mmol/L	5.52 ± 1.10	5.59 ± 1.45^b^	5.40 ± 1.17	<0.001^*^
TG, mg/dl	1.90 ± 1.46	2.01 ± 1.80^b^	1.79 ± 1.42	<0.001^*^
TC, mg/dl	4.99 ± 1.05	4.87 ± 0.94^b^	4.69 ± 0.91^c^	<0.001^*^
HDL-C, mg/dl	1.24 ± 0.34	1.15 ± 0.28^b^	1.17 ± 0.29^c^	<0.001^*^
LDL-C, mg/dl	2.65 ± 0.82	2.61 ± 0.71	2.57 ± 0.72	0.172
ALT, IU/L	24.42 ± 14.55	26.20 ± 21.67	25.97 ± 22.89	0.635
AST, IU/L	20.43 ± 6.93	20.66 ± 11.53	20.13 ± 11.03	0.324
UA, μmol/L	368.79 ± 87.17	376.16 ± 88.22^b^	355.69 ± 90.35	<0.001^*^
TSH, mIU/L	1.72 ± 1.06	1.71 ± 1.06^b^	1.81 ± 1.17	0.024^*^
FT3, pg/ml	2.82 ± 0.60	2.80 ± 0.36^b^	2.75 ± 0.36^c^	<0.001^*^
FT4, ng/dl	2.21 ± 1.05^a^	1.86 ± 0.91^b^	1.40 ± 0.73^c^	<0.001^*^
Tg-Ab, IU/ml	16.42 ± 101.36	11.13 ± 53.11	16.15 ± 70.76	0.069
TPO-Ab, IU/ml	20.13 ± 106.75	16.17 ± 81.40	17.85 ± 88.50	0.776
HT (yes)	12 (7.5%)	100 (7.7%)	354 (9.3%)	0.186^†^

*Tg-Ab, thyroglobulin antibody; TPO-Ab, thyroid peroxidase antibody*.

* *One-way ANOVA*;

† *chi-square test*.

a *Significant difference between insufficiency group and sufficiency group*;

b *significant difference between deficiency group and sufficiency group*;

c *significant difference between insufficiency group and deficiency group*.

### Analysis of the Linear Relationship Between Vitamin D and Related Variables

**Figure 3:**
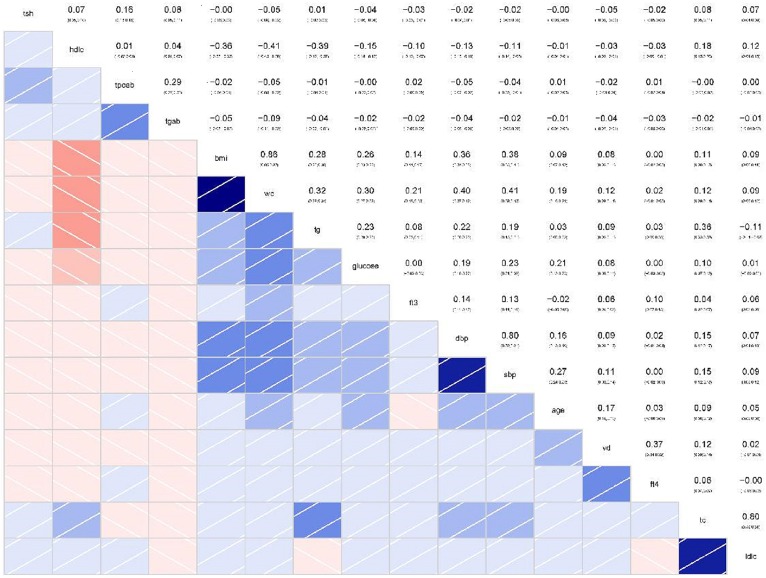
Correlations between continuous variables and correlation coefficients (Red represents a positive correlation, blue represents a negative correlation, and the darker the color, the greater the correlation coefficient).

In the correlation analysis of continuous variables, the correlation between FT4 and 2(OH)D level was the most significant (*r* = 0.37, 95% CI 0.34–0.39, *p* < 0.001). However, there was no significant correlation between 25(OH)D and TPO-Ab (r = −0.04, *p* = 1.00), or Tg-Ab and 25(OH)D (*r* = −0.04, *p* = 0.21), LDL (*r* = 0.02, *p* =1.00), or HDL (*r* = −0.03, *p* = 0.66) (Figure 3).

According to the correlation between 25(OH)D and other variables, we conducted further linear regression analysis. Linear regression analysis showed that, except for sex and age, 25(OH)D concentration was significantly correlated with BMI, waist circumference, TC, TSH, and FT4 (Table 4). This means that after adjusting for sex, age, BMI, waist circumference, and TC, 25(OH)D increased by 1 ng/ml at the normal reference level, with an increase of 2.78 ng/dl in FT4 concentration and a decrease of 0.17 mIU/L in TSH.

**Table 4:** Regression coefficient between 25(OH)D and other continuous variables.

	**B**	***p*-value**
Male gender	1.780	< 0.001
Age	0102	< 0.001
BMI	0.131	0.015
WC	−0.050	0.011
SBP	0.023	0.011
TC	0.482	< 0.001
TSH	−0.170	0.002
FT4	2.785	< 0.001

## Discussion

In our study, we analyzed data from healthy subjects who underwent the same tests. HT is the most prevalent autoimmune thyroid disorder; currently, there is no effective means of prevention and treatment of HT. HT can develop into hypothyroidism and is thought to be associated with thyroid lymphoma and papillary thyroid cancer (10). The incidence of HT has been increasing in recent years, reaching about 10 times that in the 1990s (11). Our study found a positive correlation between men and HT. The research (12) has reported a progressively increasing frequency of HT in males. Such results are more consistent with our male-positive correlation. Our study found that BMI, waist circumference, systolic blood pressure, diastolic blood pressure, blood glucose, TG, HDL, ALT, AST, UA, and TSH were significantly different between the non-HT group and the HT group, and the level of 25(OH)D was lower in the HT group. Previous studies have confirmed that vitamin D is often reduced in patients with HT (13), which is consistent with our findings. The researchers suggest that most HT patients are asymptomatic and that subclinical hypothyroidism may be present in 8% of women and 3% of men (14). Our study found that TSH increased significantly in the HT group, while no significant abnormalities were found in FT3 and FT4, indicating that HT patients were more prone to subclinical hypothyroidism.

In univariate regression analysis, we found that HT patients were positively correlated with being male, BMI, systolic blood pressure, diastolic blood pressure, waist circumference, TG, TSH, FT3, and 25(OH)D. After adjusting for known risk factors, multiple regression analysis showed that HT was statistically significantly correlated with being male, BMI, waist circumference, and TSH, but not with 25(OH)D. Therefore, sex, TSH, and other factors are independent factors of HT, while vitamin D is not. There have been a number of studies on genes and HT, and the meta-analysis indicates that the correlation between HT and gene polymorphism is more significant (15). Therefore, the significant correlation between HT and sex is more indicative of the high incidence in women, which is more due to the presence of related genes on the X chromosome, which is consistent with other research results and can be clearly explained. Studies have shown that vitamin D supplements can reduce the level of thyroid antibodies in HT patients and enhance the autoimmune function of the thyroid (16). Our study suggested that vitamin D levels were lower in the HT group, which may also explain the possibility that vitamin D can effectively reduce thyroid antibody titers. However, our study did not show a significant correlation between vitamin D and HT, so it can be seen that vitamin D may be reduced in HT, but lower vitamin D level is not a risk factor for HT, and it cannot cause an increase in its incidence. TSH is considered a potential stimulator of interleukin (IL)-6, IL-12, and tumor necrosis factor (TNF)-α in HT patients (17). Our study found that TSH was higher in the HT group, and according to the literature, TSH further promoted the release of inflammatory factors, suggesting that TSH may be associated with the occurrence of HT.

Studies in recent years have shown that the association between HT and vitamin D remains controversial. Recent studies have found no effect on vitamin D levels in the HT group compared with the control group (18). Another researcher suggested that vitamin D was not involved in the early stages of HT (19). In contrast, a case–control study found reduced vitamin D in patients with HT (20). Another meta-analysis also concluded that vitamin D deficiency was associated with autoimmune thyroid disease (21). Although our study found that 25(OH)D levels in the HT group decreased, no significant correlation was found between them by multivariate regression analysis, so we grouped them according to 25(OH)D levels. And we found that there were significant differences in thyroid hormones among the three groups; however, there was no significant difference in the prevalence of HT among the different 25(OH)D level groups.

Our study showed that TSH levels in the insufficiency group and deficiency group were higher than those in the sufficiency group, and FT3 and FT4 levels in the insufficiency group and deficiency group were lower than those in the sufficiency group. However, there was no significant correlation between 25(OH)D and thyroid antibody. Linear regression analysis showed that 25(OH)D increased by 1 ng/ml at the normal reference level, with an increase of 2.78 ng/dl in FT4 concentration and a decrease of 0.17 mIU/L in TSH. A study (18) found that the decrease of FT4 level is a predictor of vitamin D deficiency in HT patients, which is consistent with our findings, suggesting that thyroid hormone can play a role in regulating the autoimmune function of the thyroid when vitamin D is sufficient. Our study found a positive correlation between FT4 levels and 25(OH)D levels, suggesting that vitamin D supplementation or thyroid hormones may be used to regulate the balance between FT4 levels and 25(OH)D. The study confirmed that vitamin D deficiency was directly related to the short-term and long-term existence of HT, and pointed out that the severity of vitamin D deficiency was related to the duration of the disease (22). However, Ke et al. found no association of FT4 and TSH with vitamin D insufficiency in HT (23). In a cross-sectional study (24), the concentration of vitamin D was associated with TSH, and the higher the vitamin D, the lower the TSH, which was consistent with our findings. Other studies have found that taking vitamin D can reduce the titer of thyroid antibodies, especially TPO-Ab, suggesting that vitamin D may be able to affect the effect of FT4 on autoimmune diseases (25). However, our study found that there was no significant correlation between 25(OH)D level and thyroid antibody titer, which may be due to the fact that we grouped 25(OH)D levels in the selected population and not in HT patients, so the results were different among the selected population. To sum up, the incidence of HT was higher in the physical examination population, and it was more common in women. The 25(OH)D level in the HT group was significantly lower than that in the control group, but the multivariate regression analysis suggested that women and TSH were independent risk factors of HT, while 25(OH)D was not. Analysis of different 25(OH)D level groups found that TSH was negatively correlated with 25(OH)D, while FT3 and FT4 levels were positively correlated with 25(OH)D, suggesting that 25(OH)D supplementation may regulate thyroid hormone levels in HT patients. The advantage of our study is that the data volume is relatively large and more convincing. However, there are still some limitations: (1) it is a retrospective study with no further follow-up; (2) it is not possible to further clarify the normal movement conditions and light time. It is hoped that questionnaires will be designed in a planned way in future studies to investigate the diet, exercise, and light duration of the physical examination subjects, so as to further exclude vitamin D reduction caused by other reasons.

## Conclusion

Patients with HT present with reduced 25(OH)D level, and TSH is an independent risk factor for HT. TSH is negatively correlated with 25(OH)D level. FT3 and FT4 levels were positively correlated with 25(OH)D levels.

## Data Availability Statement

The datasets generated for this study are available on request to the corresponding author.

## Ethics Statement

Zhejiang University Committee waived the requirement for ethical approval for this study due to retrospective analysis, in accordance with the national legislation and the institutional requirements.

## Author Contributions

GC wrote the article. YZ did the analysis. LF guided.

### Conflict of Interest

The authors declare that the research was conducted in the absence of any commercial or financial relationships that could be construed as a potential conflict of interest.
